# Personal

**Published:** 1899-06

**Authors:** 


### PERSONAL.

Dr. Charles A. L. Reed, of Cincinnati, has resigned from the state board of medical registration and examination. The entire profession of the state will learn of this step with sincere regret as his work upon the board has been of the very greatest value. The work which he accomplished in Cincinnati alone has been something almost phenomenal. The profession loses from this position one of the most capable and energetic men whom it is possible to find in the state. Dr. Reed's reasons for this step, however, are that his own interests will no longer bear the serious sacrifice of time which has been necessitated by his successful work in that position. On April 28th, the Governor appointed Dr. A. Ravogli, of Cincinnati, to fill the vacancy made by Dr. Reed's resignation.—*Cleveland Journal of Medicine.*

---

Dr. Daniel Lewis, of the City of New York, was reëlected president of the state board of health at its annual meeting, held at Albany, May 11, 1899. This is a proper recognition of the service Dr. Lewis is rendering the state as an efficient public health officer.

---

Dr. Floyd S. Crego, who is abroad on account of ill-health, is reported to be improving rapidly. He expects to spend the summer in Heidelberg and to return to Buffalo in the early autumn when he will resume the practice of his profession.

---

Dr. John Dodds Flagg, of Buffalo, announces his removal from 322 Pennsylvania street to the Wellesley, corner of Franklin and Edward streets. Practice limited to diseases of the eye and ear. Hours: 9 a. m. to 1 p. m.

Dr. A. B. Allen, of Buffalo, begs to announce the removal of his office and residence from 962 Fillmore avenue, to 413 East Utica street, near Jefferson. Hours: 9.30 a. m., 1 to 3 and 7 p. m.

---

Dr. N. L. Burnham, formerly resident physician Buffalo General Hospital, has established his office at 478 Franklin street. Hours: 8 to 9 a. m.; 2 to 3 and 6 to 7 p. m. Telephone.

---

Dr. Andrew F. Currier, of New York City, has moved his office from 120 East Thirty-fourth street to 113 East Fortieth street. Hours: 11 to 1. Telephone, 1025, 38th.

---

Dr. William C. Krauss, of Buffalo, will sail for Europe, June 13, 1899, for a six weeks' tour of recreation. Mrs. Krauss will accompany him.

---

Dr. John B. Deaver, of Philadelphia, has resigned as assistant professor of applied anatomy at the University of Pennsylvania.

---

Dr. George F. Cott, of Buffalo, has removed from 1302 Michigan street to 16 Woodlawn avenue.

---

Dr. George S. Palmer, of Buffalo, has removed from 280 Franklin street to 325 Franklin street.

